# Partial Thymectomy during Tracheostomy for Superior Herniation of Normal Mediastinal Thymus in a Patient with Larsen Syndrome: A Case Report

**DOI:** 10.70352/scrj.cr.24-0158

**Published:** 2025-05-09

**Authors:** Atsushi Yoshiyama, Kaori Morita, Shinya Takazawa, Motoki Ebihara, Mitsuharu Yahiro, Tomo Kakihara, Mariko Yoshida, Jun Fujishiro

**Affiliations:** Department of Pediatric Surgery, The University of Tokyo Hospital, Tokyo, Japan

**Keywords:** thymus, herniation, thymectomy, tracheostomy, Larsen syndrome, connective tissue disorder

## Abstract

**INTRODUCTION:**

Superior herniation of the mediastinal thymus is a rare cause of neck mass, characterized by intermittent migration of normal thymic tissue into the suprasternal region due to increased intrathoracic pressure. Generally, thymus resection is discouraged to avoid inducing athymia and subsequent immunodeficiency in the child. To date, no prior cases of tracheostomy combined with partial thymectomy have been reported. We present a case in which partial resection of the thymus was necessary to facilitate a tracheostomy.

**CASE PRESENTATION:**

A 3-month-old female infant diagnosed with Larsen syndrome, a rare congenital connective tissue disorder, presented with respiratory failure necessitating mechanical ventilation at birth. Despite successful extubation and subsequent management with noninvasive positive pressure ventilation, she experienced recurrent episodes of apnea and oxygen desaturation. Examination revealed an anterior midline neck swelling, synchronized with respiratory movements, originating from the suprasternal notch. Ultrasound, computed tomography, and magnetic resonance imaging of the neck confirmed the presence of a normal mediastinal thymus extending into the suprasternal region. Given the risk of upper airway stenosis after the otolaryngological evaluation, an early tracheostomy under general anesthesia was planned. Upon incising the thickened cervical fascia, the thymus was visualized on the anterior surface of the trachea. The thymus and its surrounding adhesions were separated, with resection of the upper pole, followed by closure of the hernia orifice. The tracheostomy was then performed as planned. The postoperative course was uneventful, marked by gradual respiratory improvement and resolution of the intermittently visible swelling during inspiration. Cannula exchanges were completed without complications, and the patient was discharged home with a heat moisture exchanger 3 months after surgery.

**CONCLUSIONS:**

We encountered a case of superior herniation of the normal mediastinal thymus in a patient with Larsen syndrome. While speculative, connective tissue abnormalities may contribute to this condition. In cases requiring tracheostomy, partial thymectomy and closure of the hernia orifice may be necessary to maintain fistula patency postoperatively.

## Abbreviation

*FLNB*
: filamin B

## INTRODUCTION

Superior herniation of the normal mediastinal thymus is a rare neck mass characterized by the intermittent migration of thymic tissue into the suprasternal region, driven by increased intrathoracic pressure, such as that occurring during breathing.^1)^ Generally, thymus resection is avoided to prevent athymia and subsequent immunodeficiency in the child.^2)^ However, partial thymectomy may be required during tracheostomy. Currently, no previous reports describe tracheostomy combined with partial thymectomy. Here, we present a case in which resection of the normal thymic tissue was necessary to perform a tracheostomy.

## CASE PRESENTATION

A neonate, the firstborn of a dichorionic-diamniotic twin pregnancy, was delivered via cesarean section at 36 weeks and 5 days gestation. The presence of multiple joint contractures, bilateral hip dislocation, and facial anomalies suggested Larsen syndrome, a congenital connective tissue disorder. Fetal magnetic resonance imaging revealed airway narrowing, and after birth, the infant displayed irregular spontaneous breathing with marked retractions. Resuscitation with mask ventilation and continuous positive airway pressure was successful, and the infant was subsequently admitted to the neonatal intensive care unit. Due to persistent intercostal retractions and suspected upper airway stenosis upon admission, endotracheal intubation was performed. As respiratory status stabilized, the infant was extubated on day 12 and managed with nasal-directional positive airway pressure to prevent apnea. Despite this, frequent episodes of oxygen desaturation and apnea continued. An otolaryngological evaluation revealed bilateral arytenoid mucosal swelling, leading to laryngeal stenosis, along with severe dysphagia, significantly increasing the risk of aspiration. Consequently, an early tracheostomy was deemed necessary.

**Fig. 1:**
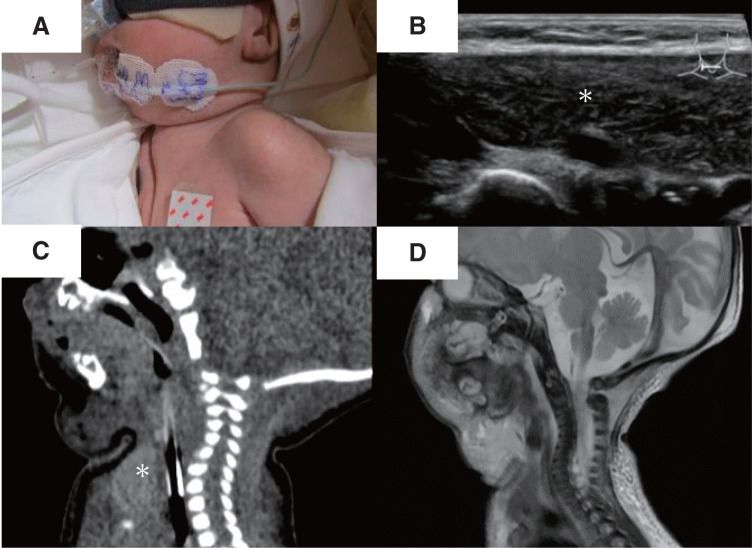
(**A**) A mass intermittently visible at the midline of the neck during exhalation, particularly when crying. (**B**) Axial ultrasonographic image of the suprasternal region, showing the thymus (∗) protruding superiorly into the neck. (**C**) Sagittal CT image and (**D**) T2-weighted MR image, both showing slight cephalad protrusion of the thymus (∗). CT, computed tomography; MR, magnetic resonance

**Fig. 2:**
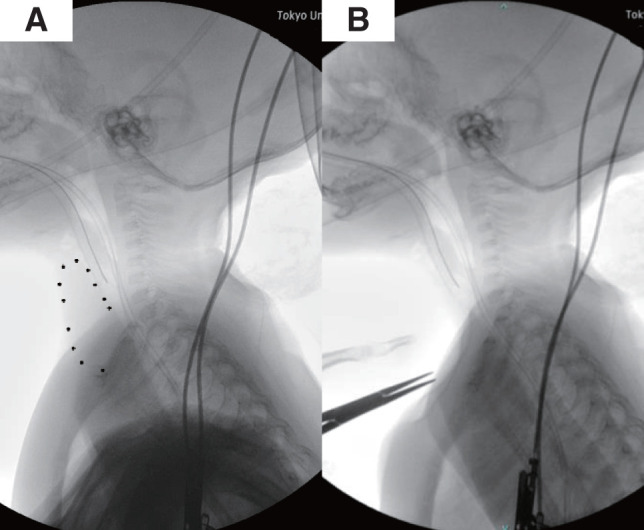
(**A**) Preoperative fluoroscopic examination of the neck in lateral view. (**B**) The thymus was manually displaced posterior to the sternum.

Before the tracheostomy, a mass intermittently visible at the midline of the neck during exhalation, especially when crying, was noted (**Fig. 1A**). Neck ultrasound revealed a homogeneous hypoechoic structure with internal echogenic foci extending from the retrosternal region to the inferior border of the thyroid gland (**Fig. 1B**). Sagittal sections of plain computed tomography and T2-weighted magnetic resonance imaging showed a slight extension of the upper pole of the thymus into the neck (**Fig. 1C** and **1D**). A preoperative fluoroscopic image demonstrated manual displacement of the thymus posterior to the sternum (**Fig. 2A** and **2B**).

**Fig. 3:**
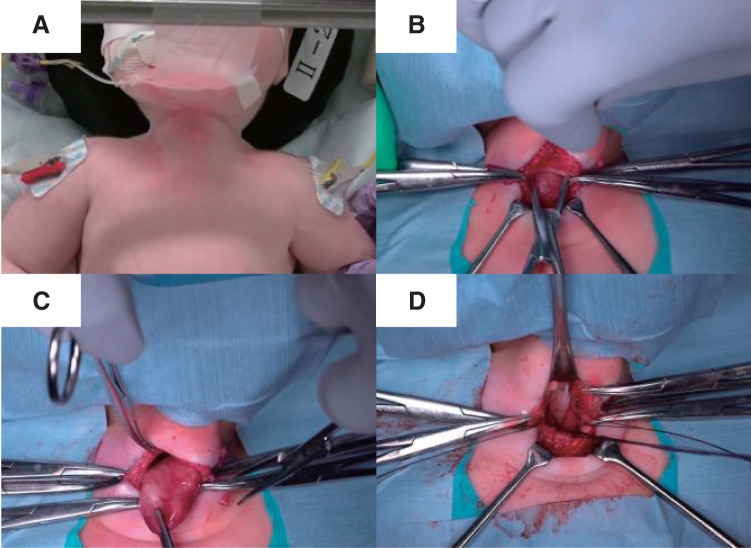
Operative procedure. (**A**) The mandible was retracted to extend the neck. (**B**) Thymic tissue was identified at the anterior surface of the trachea after dissecting the midline tissue. (**C**) Adhesions with surrounding tissues were dissected, allowing for mobilization of the thymus. (**D**) After resecting the cephalad portion of the thymus, the hernia orifice was closed, exposing the anterior surface of the trachea.

**Fig. 4:**
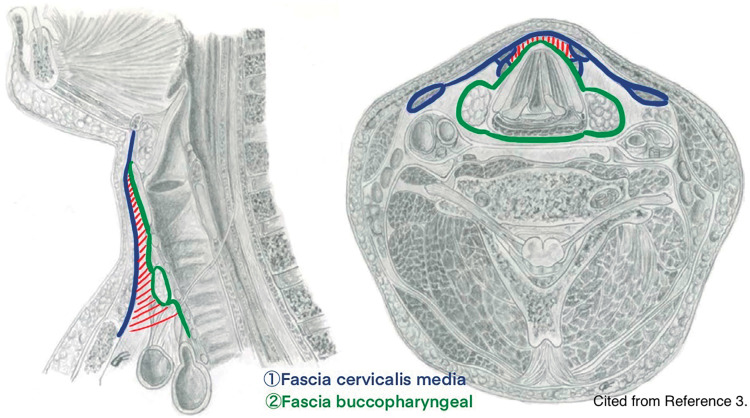
In the schematic diagram (cited from reference 3), the fascia cervicalis media and fascia buccopharyngeal are depicted in blue and green, respectively. The thymus was herniating into the hatched area enclosed by these 2 fascial layers.

We performed a tracheostomy with partial thymectomy. The patient was positioned supine under general anesthesia, with the neck hyperextended by retracting the mandible (**Fig. 3A**). Under positive pressure ventilation, the respiratory fluctuations of the thymus ceased, and it remained consistently prolapsed from the mediastinum. The thymus, which appeared to protrude with respiration, was palpable in front of the trachea, obstructing palpation of the cricoid cartilage. A midline incision was made in the thickened fascia cervicalis media, likely due to friction-induced localized fibrous proliferation, and thymic tissue was revealed (**Figs. 3B** and **4**). The thymus was loosely adherent to the surrounding tissue. After separating these adhesions to mobilize the thymus (**Fig. 3C**), the upper pole was resected using LigaSure (Medtronic, Minneapolis, MN, USA). The thymus appeared as a single mass without distinct right and left lobes. As it was excised using LigaSure, no arterial or venous structures were identified. The resection rate was estimated to be approximately 30%–50% based on intraoperative visual assessment of the remaining thymic tissue and the size of the excised specimen. The thickened fascia cervicalis media and fascia buccopharyngeal^3)^ were sutured with 3-0 absorbable suture to close the hernia orifice (**Fig. 3D**). Following partial thymectomy, cranial extension of the incision in the thickened fascia cervicalis media exposed the thyroid gland. The tracheostomy was then performed as planned. The procedure took 60 min with minimal blood loss. Although fragile connective tissue raised concerns about inflammation spreading to the mediastinum, the surgery was not performed for an inflammatory disease. Thus, preventive measures for postoperative mediastinitis were limited to perioperative prophylactic antibiotics (sulbactam/ampicillin) and meticulous hemostasis. Histopathological examination of the resected specimen confirmed normal thymic tissue, measuring 32 × 30 × 12 mm. The postoperative course was uneventful, with gradual respiratory improvement. The first cannula exchange, performed 2 weeks postoperatively, was without complications. Approximately 3 months after surgery, the patient was discharged home, requiring only a heat moisture exchanger without ventilator assistance. The patient was readmitted with a viral upper respiratory tract infection 1 week after discharge. Approximately 6 months postoperatively, respiratory deterioration due to intratracheal granulation tissue from cannula tip pressure required hospitalization for cannula adjustment. However, no increased susceptibility to infections has been observed.

## DISCUSSION

Superior thymic herniation is a rare condition, with only a few reported cases.^1)^ It should be considered in the differential diagnosis of an intermittent neck mass palpable only during increased intrathoracic pressure. Literature suggests that thymic herniation can be diagnosed via ultrasound. In the absence of respiratory compromise, such as tracheal compression, invasive interventions—including biopsy, radiation, or surgery—should be avoided in favor of conservative management.^4)^

We present a case of superior herniation of the normal mediastinal thymus in a 3-month-old female infant. Stuut et al. suggested that thymic herniation may be associated with local loose connective tissue.^5)^ In our case, the patient also had the rare congenital connective tissue disorder Larsen syndrome, caused by mutations in the *FLNB* gene, which plays a key role in cytoskeletal regulation. Patients with Larsen syndrome present with physical characteristics such as large-joint dislocations and craniofacial anomalies, both observed in our patient. Additionally, respiratory deterioration requiring ventilatory support may occur due to spinal cord compression from conditions like kyphoscoliosis.^6)^ Although thymic herniation has not been previously reported in association with Larsen syndrome, it is plausible that connective tissue disorders may contribute to the development of thymic hernia.

As the thymus is naturally replaced by adipose tissue after puberty, the neck mass is expected to either regress or, at the very least, not increase in size.^7)^ Consequently, unless adjacent structures are compromised, medical or surgical intervention is typically unnecessary.^8,9)^ However, in our case, a tracheostomy was required for long-term respiratory management due to upper airway obstruction, and there was concern that the thymus could interfere with the procedure. Indeed, during surgery, the thymus obstructed the surgical field, necessitating either its reduction or repositioning. Given that returning the thymus and closing the hernia orifice could increase intrathoracic pressure and adversely affect hemodynamics, we opted for partial resection. To prevent postoperative complications related to the cannula, we also closed the hernia orifice. Although no previous cases of tracheostomy in thymic hernia exist for comparison, we believe the surgical approach we employed was effective.

## CONCLUSIONS

We encountered a case of superior herniation of the normal mediastinal thymus. While speculative, connective tissue abnormalities may contribute to this condition. In cases where tracheostomy is required, partial thymectomy and hernia orifice closure may be considered to ensure postoperative fistula patency.

## DECLARATIONS

### Funding

No funding was received.

### Authors’ contributions

ST, KM, and MY examined and managed the patient.

AY drafted the manuscript.

ST revised it critically for important intellectual content.

JF gave final approval for the content.

All authors read and approved the final manuscript.

All authors have agreed to take responsibility for all aspects of the research.

### Availability of data and materials

The dataset supporting the conclusions of this article is included within the article.

### Ethics approval and consent to participate

Not applicable as a case report is not regarded as a study according to the “Ethical Guidelines for Medical and Health Research Involving Human Subjects” of the Japanese Ministry of Health, Labour and Welfare. The patient has provided informed consent for participation in the study.

### Consent for publication

Written informed consent was obtained from the patient’s guardian for publication of this case report, including their medical data and images.

### Competing interests

The authors declare that they have no competing interests.
